# Exquisite Light Sensitivity of *Drosophila melanogaster* Cryptochrome

**DOI:** 10.1371/journal.pgen.1003615

**Published:** 2013-07-18

**Authors:** Pooja Vinayak, Jamie Coupar, S. Emile Hughes, Preeya Fozdar, Jack Kilby, Emma Garren, Taishi Yoshii, Jay Hirsh

**Affiliations:** 1 University of Virginia, Department of Biology, Charlottesville, Virginia, United States of America; 2 Graduate School of Natural Science and Technology, Okayama University, Okayama, Japan; Queen Mary, University of London, United Kingdom

## Abstract

*Drosophila melanogaster* shows exquisite light sensitivity for modulation of circadian functions *in vivo*, yet the activities of the *Drosophila* circadian photopigment cryptochrome (CRY) have only been observed at high light levels. We studied intensity/duration parameters for light pulse induced circadian phase shifts under dim light conditions *in vivo*. Flies show far greater light sensitivity than previously appreciated, and show a surprising sensitivity increase with pulse duration, implying a process of photic integration active up to at least 6 hours. The CRY target timeless (TIM) shows dim light dependent degradation in circadian pacemaker neurons that parallels phase shift amplitude, indicating that integration occurs at this step, with the strongest effect in a single identified pacemaker neuron. Our findings indicate that CRY compensates for limited light sensitivity *in vivo* by photon integration over extraordinarily long times, and point to select circadian pacemaker neurons as having important roles.

## Author Summary

We investigate the paradox that fruit flies show exquisite light sensitivity for day/night circadian clock functions, yet the circadian photoreceptor cryptochrome (CRY) responds only to very high light levels in assays requiring immediate responses. Our *in vivo* behavioral assays are unique in that we expose flies to dim and limiting levels of light. We find that CRY integrates photons efficiently over time periods of at least six hours, with light sensitivity unexpectedly increasing with duration of light exposure. This contrasts with image-forming responses that occur on millisecond time scales in *Drosophila*. We show that light dependent degradation of the CRY target timeless (TIM) occurs at limiting light levels, closely paralleling behavioral effects, in the circadian pacemaker neurons. One of these neurons shows particularly strong light sensitivity, and a particularly strong temporal integration effect. We have thus identified the precise step at which temporal integration is functioning. The structurally unrelated vertebrate circadian photoreceptor melanopsin also shows the ability to integrate photons over time, though not to the extent of *Drosophila* CRY. We thus conclude that temporal integration is a universal mechanism to enhance photosensitivity of non-visual photopigments.

## Introduction

Nearly all plants and animals use daily patterns of day and night to entrain their endogenous circadian oscillators. These responses utilize photic input from both visual photoreceptors, as well as from non-visual circadian photopigments (reviewed in [1]–[3]). Both visual photopigments and the circadian blue light photopigment cryptochrome (CRY) are required in *Drosophila* for normal entrainment to a light/dark cycle, but CRY is the sole photopigment required to shift circadian phase after a light pulse given in subjective night, and flies are circadian blind when both cryptochrome and visual photopigments are absent [4]–[7]. Additionally, CRY is the photopigment leading to behavioral arrhythmicity in response to constant light [8]. In addition to these light input pathways, another less well defined pathway involves the developmental gene *glass* [4], [9], [10], with a role in light/dark entrainment.

CRY has two circadian roles in *Drosophila*. In the core circadian oscillator, it functions to trigger light dependent ubiquitinylation and degradation of its target timeless (TIM), a core circadian factor, as well as itself [11]. In cells peripheral to the central oscillator, CRY functions as a transcriptional repressor of CLK/CYC, binding to the PER protein, a role similar to its role in mammals [12] in addition to its role in making most fly tissues inherently light sensitive [13]. A pathway has been worked out for the light signaling of CRY in *Drosophila* through a large series of studies (reviewed in [2], [3]). To summarize briefly, CRY binds TIM following a light dependent conformational change. This then triggers the Jetlag dependent degradation of both CRY and TIM, with more rapid degradation of TIM vs CRY based on enhanced affinity of JET for TIM [14].

Flies are extremely light sensitive for circadian clock entrainment [15], responding to less than 0.03 nw/cm^2^ 12 hr ‘days’ of blue light in an LD cycle. Half-maximal shifts of circadian phase resulting from a light pulse during late subjective night can result from a 20–30 µw/cm^2^,10 minute white light pulse [16], [17] Half-maximal shifts in phase of eclosion timing occur at blue light intensities of 100 nw/cm^2^ (3×10^11^ photons/cm^2^/sec) in a related *Drosophilid* [18]. However, physiological and biochemical responses of CRY to light are observed at far higher intensities, at or above 1 mw/cm^2^. These include a light induced conformational change [19], light induced ubiquitinylation of the TIM protein and subsequent CRY degradation [11], and light induced stimulation of neuronal firing rate, either in normal CRY containing neurons, or in neurons with ectopic CRY expression [20]. This discrepancy indicates that some process must be operative *in vivo* to increase the effective light sensitivity of CRY, or that the high light intensity responses of CRY may not be relevant to its *in vivo* function.

Here we investigate the discrepancy between the low light sensitivity of CRY for its measured activities, relative to the extreme light sensitivity for its *in vivo* phase shifting effects. We measure the half-maximal responses of flies to a late subjective night light pulse, varying both light intensity and duration, and find far greater light sensitivity than previously appreciated. We find a surprising intensity vs duration relationship, with increasing phase shift amplitude as photon number is held constant with increased light pulse duration. This implies an ability to integrate photon information over durations of hours that is almost exclusively dependent on CRY photic input. We then show that these photon-limited responses lead to TIM degradation, with significantly more TIM degraded by an equal-photon-number long duration light pulse. This indicates that temporal integration increases efficacy of TIM degradation. These observations provide a general means by which a low-sensitivity photopigment can achieve extraordinarily high effective light sensitivity.

## Results

**Figure 1:**
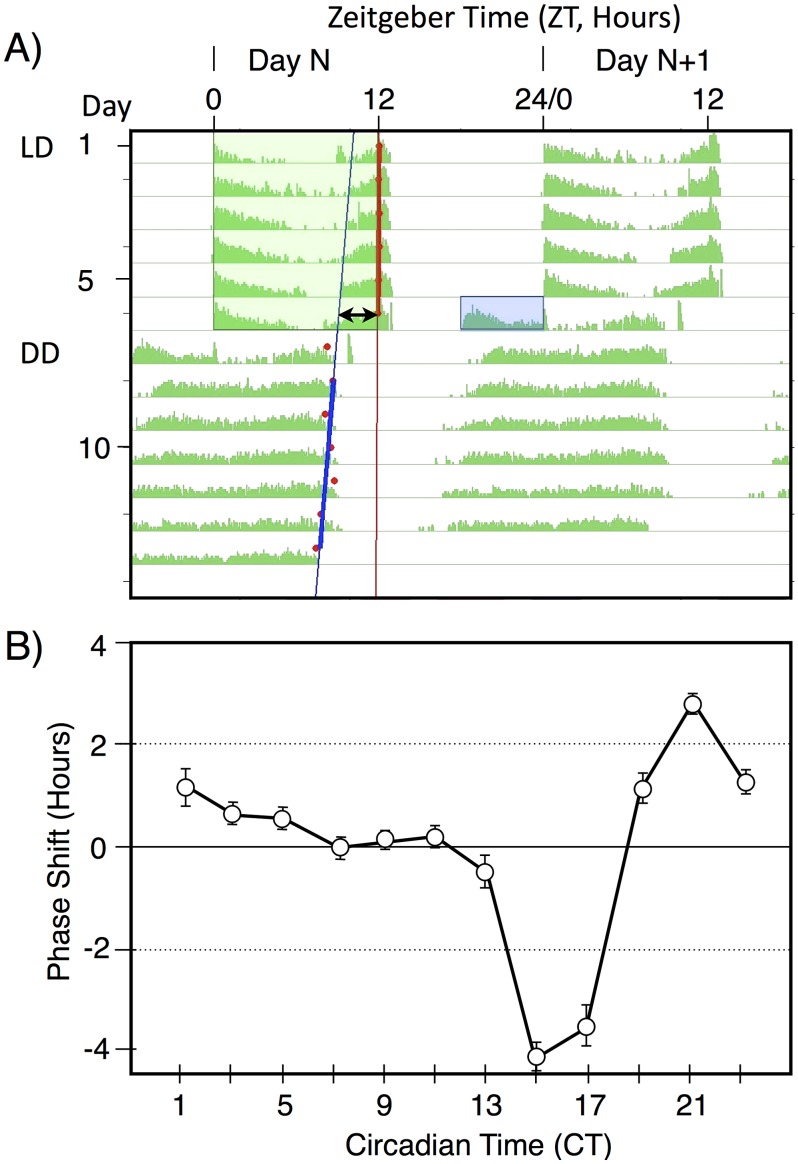
A double-plotted median actogram illustrating the basic paradigm used in this manuscript (A), and a Phase Response Curve (B). A) A median actogram showing the results of a light pulse during late subjective night on circadian phase in constant darkness (DD). Flies were entrained to a 12 hr;12 hr light dark schedule with green light for 6 days, then given a 6 hr blue light pulse of intensity 33 nw/cm^2^ at ZT18-24. The resulting phase advance (double arrow line) is shown by extrapolating from the computer called RMS line (blue) marking the activity off times (red dots) in DD. Phase shift data in this manuscript is computed from individual flies, relative to the phase shift of a control set of flies not receiving a light pulse. Data in this figure is double plotted for ease of viewing, but annotations are only shown for one of the two occurrences. B) A phase response curve, showing the averaged phase shifts resulting from a 1 hr light pulse at the indicated circadian times. Light pulses were of white light of intensity ∼150 µw/cm^2^. Averaged data reprinted from [24].

The basic light pulse paradigm used in this manuscript is illustrated in Figure 1A. This figure shows a median actogram derived from 12 individual flies. Flies were entrained to a 12 hr∶12 hr light/dark schedule (green box), then given a 6 hr exposure to a pulse of blue light (blue box) late in subjective night at ZT18-24. This light pulse stimulates activity within the flies subjective night and leads to a phase advance, as shown by the double arrow line. This line compares circadian phase in LD, taken as the lights off point, versus the extrapolated activity off points in constant darkness, with the blue line derived from RMS match to the red activity off points. Light pulses late in subjective night result in phase advances, whereas light pulses early in subjective night result in phase delays (Figure 1B). In this manuscript we restrict our analyses to the phase advance region of the phase response curve, centering light pulses around the maximum phase advance at ZT20-21.

**Figure 2:**
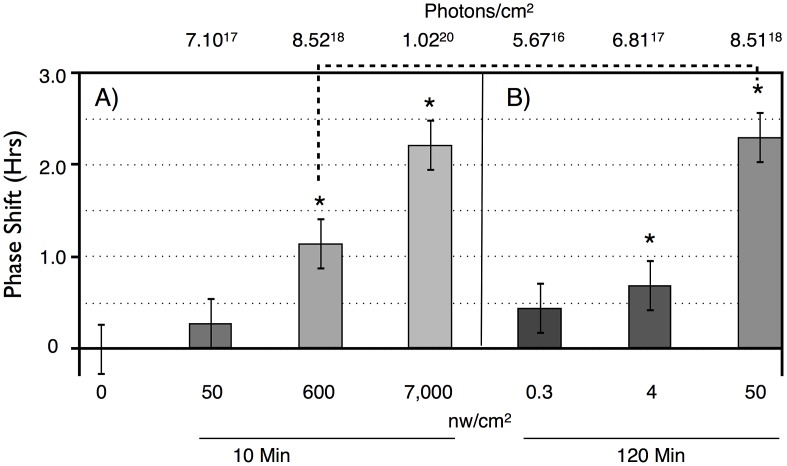
Phase shift magnitude as a function of intensity and duration of blue light pulses. LD-entrained flies were given light pulses of either: A) 10 min, or B) 120 min, at different intensities at times centered at ZT20. n = 10 per condition. For both duration light pulses, there are graded phase shift responses as a function of increasing light intensity. 10 min: 7,000 vs 50 nw/cm^2^, P = 0.02; 120 min: 50 vs 4 nw/cm^2^: P = 0.04; 4 vs 0.3 nw/cm^2^: P = 0.03 (ANOVA). Asterisks indicate significantly different values from the no pulse control. 10 min: 600 nw/cm^2^ vs no pulse: P = 0.02; 7,000 nw/cm^2^ vs no pulse: P = 0.007. 120 min: 0.3, 4 nw/cm^2^ vs no pulse: not significant; 50 nw/cm^2^ vs no pulse: P = 0.0.006. Error bars = SEM. The dashed line indicates two light pulses with equal numbers of photons: not significantly different.

To determine light sensitivity for circadian phase shifting by light pulses in subjective night, we determined half-maximal light sensitivity for blue light pulses centered at ZT20 for two time intervals, 10 min (Figure 2A), and 120 min (Figure 2B). To account for the extra time of illumination in the 120 min pulses, levels were adjusted 12× lower than for the 10 min pulses. Flies responded with graded phase advances to the 10 min pulses, with a significant phase advances at or above 600 nw/cm^2^. The 2.4 hr phase advance resulting from the 7,000 nw/cm^2^ pulse is saturating, because higher intensities do not result in a larger phase advance [16], [17]. Our half-maximal light intensity values are significantly lower, i.e., more light sensitive, than published data for ZT21 light pulses [16], [17], but the published data uses white light, whereas our monochromatic blue light is better matched to the light sensitivity of CRY for its phase shifting activity [21].

Flies show greater light sensitivity in response to the 120 min light pulses, with 4 nw/cm^2^ showing slightly less than a half-maximal advance. The enhanced light sensitivity in response to the 120 min vs 10 min light pulses can also be seen in a comparison of two datapoints in which flies received equal numbers of photons, the 600 nw/cm^2^ 10 min light pulse, versus the 50 nw/cm^2^ 120 min light pulse (dashed line in Figure 2). Though these phase advances are not significantly different (P = 0.11, ANOVA), the latter is certainly not smaller than the former. This shows the possibility of a surprising intensity vs duration relationship, suggesting that flies might utilize a mechanism that allows them to integrate photon information over long time intervals.

**Figure 3:**
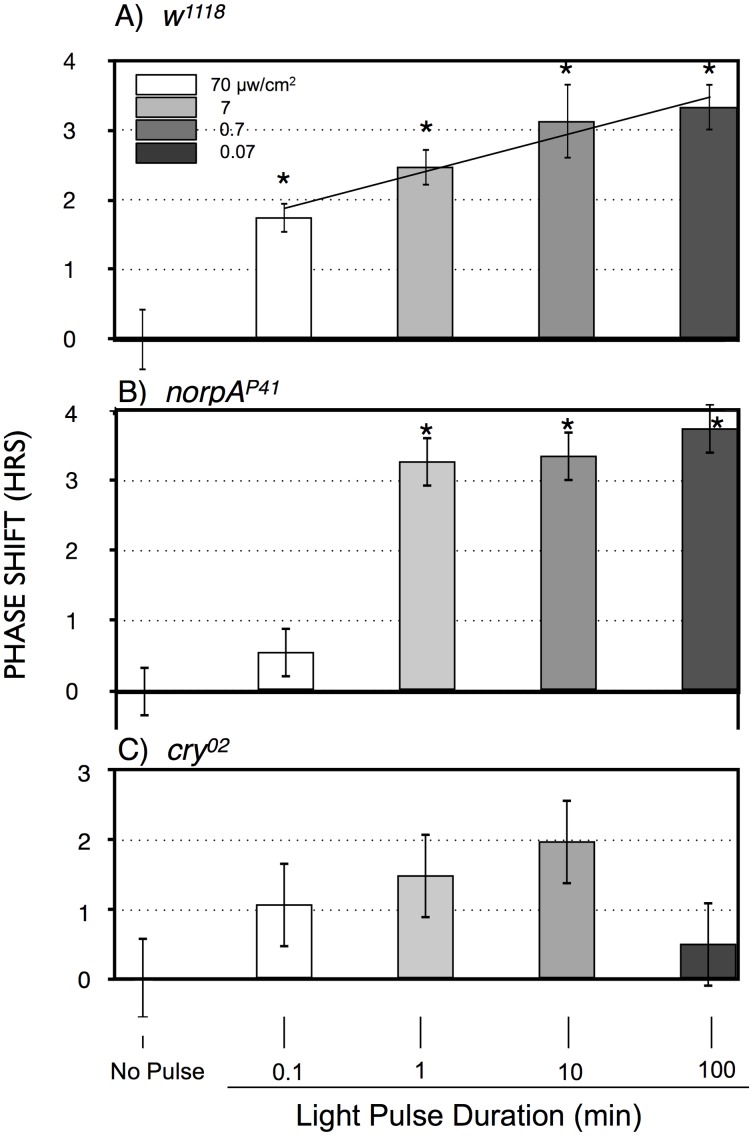
Phase shift magnitude as a function of blue light pulses with equal numbers of photons, reciprocally varying time and intensity. A) *w^1118^*; B) *norpA^P41^*; C) *cry^02^* LD-entrained flies were given light pulses of indicated duration centered at ZT20. Light intensities were controlled such that all flies received the same number of photons. Statistics, ANOVA, with post-hoc correction for multiple comparisons; n = 16–20 for each light pulse. A) All phase shifts were significant relative to the no pulse control (alpha <0.0125). The line through the light pulse points is a linear regression through the light pulse values (R^2^ = 0.14), showing a significant positive slope of 0.55±0.13 (P = 4×10^−5^), using an X scale of log_10_ light pulse duration. B) The 1, 10 and 100 min light pulses are all highly significantly different relative to the no pulse control or to the 0.1 min light pulse (P<10^−8^). C) Only the 100 min point approaches significance relative to the no pulse control (P = 0.018, alpha = 0.0125). Asterisks indicate significantly different values from the no pulse controls. Error bars = SEM.

**Figure 4:**
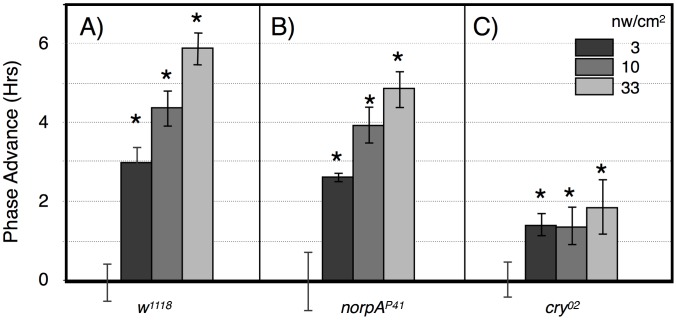
Large amplitude phase advance responses to 6 hr ZT21-centered light pulses depend primarily on CRY. A) *w^1118^* n = 15–18 per condition. B) *norpA^P41^*; n = 6–8 per condition. C) *cry^02^* n = 7–12 per condition. Asterisks: significant phase advances relative to the no pulse control (ANOVA, significance set at alpha = 0.017). Error bars = SEM.

To explicitly investigate temporal integration, we performed an experiment in which photon number was held constant as light intensity and time of a ZT20-centered blue light pulse was varied reciprocally in log increments over 3 log units (Figure 3A). This results in a graded increase in phase advance amplitude as light pulse duration increases. This indicates that phase advances in response to light pulses with equal numbers of photons are larger when light pulses are administered over 100 min as compared to 0.1 or 1 min (P<0.001; P = 0.027, respectively, ANOVA), which would imply an ability to integrate and stably store photon information for up to 100 min. As we will show later in Figure 4, light sensitivity is further enhanced for 360 min light pulses, indicating that this process occurs efficiently over times of several hours.

The two possible photic input pathways to the *Drosophila* circadian clock are the circadian photoreceptor cryptochrome, CRY, and the opsin visual photoreceptors in the eye. In Figure 3B we examined phase advances in response to equal-photon-number ZT20 centered light pulses in *norpA* flies, with severely compromised vision due to a *norpA^P41^* null mutation in the visual specific phospholipase C [22], [23]. The responses are strikingly similar to wild type flies at the three longer time intervals, indicating that the long duration photic integration is independent of the visual photoreceptors, and must be due to CRY mediated photoreception, and/or PLC-ß independent opsin signaling from eyelet [23]. The one difference between the two strains occurs at the shortest duration/highest intensity time point, where *w^1118^* but not *norpA* shows a significant phase advance (P = 0.006). This indicates that CRY photoreception functions more efficiently with longer durations and lower intensities of light than visual photoreception. To confirm this, in Figure 3C we tested *cry* null flies, *cry^02^*, in the same paradigm. As expected, these flies show highly muted phase advance amplitudes [24], [25], with none of the light pulses resulting in a statistically significant phase advance relative to the no pulse control. Comparing the data in Figure 3 for each light pulse time as a function of genotype, only the 100 min light pulse for *cry* is significantly different from *w^1118^* or *norpA* (P<0.001, ANOVA). Thus, *cry* null flies retain at most a minimal capability for temporal integration for a duration of no more than 10 min, which must be due to visual photoreceptors. Repetition of this experiment utilizing *cry^02^* at 10× or 100× (Figure S2) the intensities used in Figure 3C similarly resulted in no statistically significant phase shifts relative to the no pulse controls, indicating that light sensitivity of *cry^02^* for circadian phase shifts is at least 100× reduced relative to wild type flies (Figure 3A). As such, we turned to another assay to investigate the role of CRY vs visual photoreception in light pulse induced phase shifts.

The alternative assay examines phase advances in response to graded intensities of 360 min intervals of blue light centered at ZT21, using this time point instead of ZT20 to avoid going into the phase delay region of the phase response curve [24], [25]. Wild type flies show a graded response to increasing blue light illumination, with a half-maximal advance at 3 nw/cm^2^, and large amplitude 6 hr phase advances at 33 nw/cm^2^ (Figure 4A). This amplitude is saturating as shown using a similar though not identically timed 360 min light pulse [24]. We performed similar studies using *norpA* (Figure 4B) and *cry^02^* (Figure 4C). *NorpA* flies show phase advance responses that are indistinguishable from *w^1118^* except possibly at the highest intensity, indicating that visual phototransduction plays virtually no role in these responses. In contrast, the responses in *cry^02^* flies are severely muted, showing a ∼1.5 hr phase advance at all intensities. Similarly muted phase shift responses have long been seen for *cry* mutants [21], [24], [25], with [24] showing significant phase shifts at ZT21 and 23.

The results from this light pulse phase advance study indicate that phase advances use both CRY and visual photoreceptors, but that the major contributor to phase advance amplitude is from CRY. The lack of significant variation in phase advance amplitude as a function of intensity in *cry^02^* is most readily interpreted as showing a high sensitivity but low amplitude response, i.e., that the light intensity would need to be reduced further in order to show a half-maximal response, due to visual photoreceptors. This is consistent with the function of the opsin visual photoreceptors as extremely efficient light gathering pigments, responding to single photons by virtue of dense packaging in specialized visual structures and coupling to highly efficient downstream signaling systems [26]. In contrast, the contribution from CRY photoreception, as observed in the visually compromised *norpA* flies, has inherently low light sensitivity, but this pathway is far more effective in promoting large amplitude phase advances.

**Figure 5:**
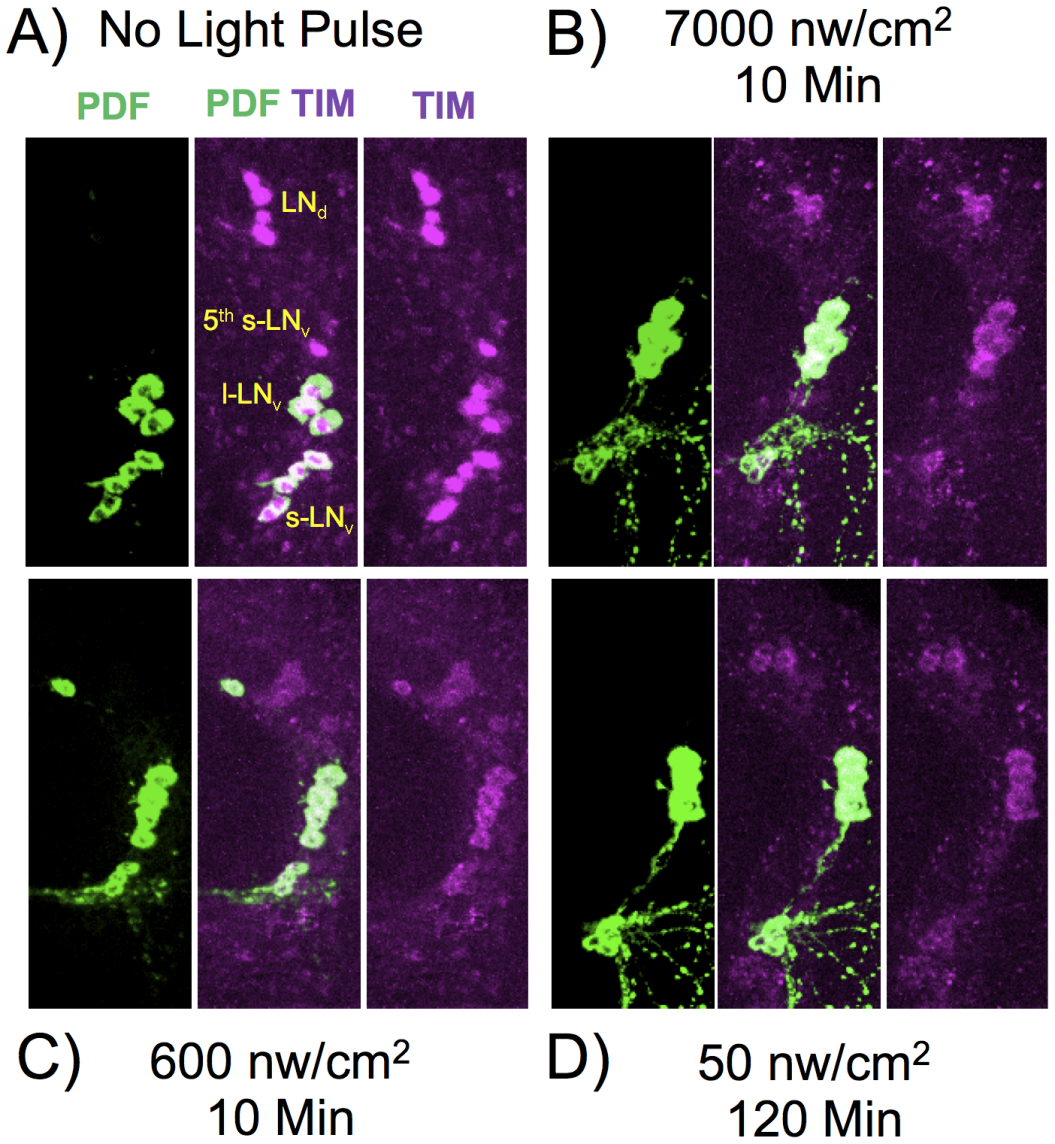
TIM and PDF immunostaining in various LNv neurons. Flies were subject to light pulses at initiating at ZT20 and assayed at ZT22. Blue light pulse conditions: A) No light pulse control. B) 7000 nw/cm^2^, 10 min. C) 600 nw/cm^2^ 10 min. D) 50 nw/cm^2^ 120 min. Four of the five s-LNv neurons are labeled with PDF as well as TIM. The 5^th^-sLNv is identified by TIM immunoreactivity and by position and morphology.

**Figure 6:**
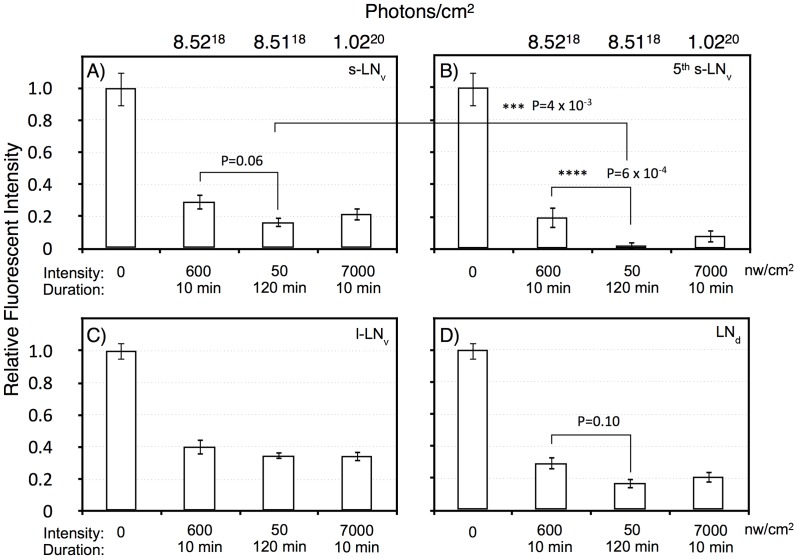
Quantitation of TIM levels in the specific LN subsets as shown in Figure 5. A) s-LN_v_. B) 5^th^ s-LN_v_ (PDF negative). C) l-LN_v_. D) LN_d_. Intensity/durations were designed based on the data in Figure 2. For each neuronal subset, we show the no light pulse control (0), 600 nw/cm^2^ 10 min, representing a roughly half-maximal intensity-duration; 50 nw/cm^2^ 120 min, an equal-photon exposure to the former, and 7,000 nw/cm^2^, 10 min, yielding a stronger phase shift. All neuronal classes show highly significant TIM degradation in response to light pulses relative to the no pulse control (P<10^−6^). The PDF negative 5^th^ s-LN_v_ was visible in 10 of 11 LNv clusters in the no pulse control, and the absolute intensity of TIM IR in this neuron was indistinguishable from the s-LN_v_ or any of the other neuronal clusters: 209±21 (SEM, arbitrary fluorescence units), vs 192±19 for the other s-LN_v_. Thus, the near disappearance of TIM immunoreactivity in the 5^th^ s-LN_v_ in response to the 50 nw/cm^2^ 120 min light pulse cannot be explained by differential detection sensitivity. This neuron showed TIM immunoreactivity in 6 of 11 LN_v_ clusters following the 600 nw/cm^2^ 10 min light pulse, but only 1 of 11 following the 50 nw/cm^2^ 120 min light pulse. Statistics were performed using two way ANOVA on square root transformed data to better approximate normality (Stat Plus, Analystsoft). Two way ANOVA values for the interaction of specific light pulse x cell types are shown in Table S1. The six LNd neurons are heterogeneous, with only three showing detectable CRY immunoreactivity [34], but our analyses do not detect a subset showing differential TIM degradation.

The phase shifting effect of a light pulse during subjective night signals via CRY dependent degradation of the core circadian gene product TIM (reviewed in [2]). To determine whether TIM degradation parallels phase shift amplitude at limiting light levels, we measured TIM levels following dim light pulses, covering the critical intensities/durations as determined in Figure 2. Since we measure TIM levels in whole mount brain preparations, we examined responses in particular clusters of brain circadian neurons, as shown in Figure 5, with quantitation by immunofluorescence in Figure 6. In this experiment, we subjected flies to ZT20 light pulses at a half-maximal level of 10 min, 600 nw/cm^2^, an equal photon dose of 50 nw/cm^2^ 120 min, and a saturating phase shifting pulse of 7000 nw/cm^2^ for 10 min, measuring levels of TIM IR at ZT22.

All the light pulse conditions show a major effect on TIM levels versus the no pulse control (two way ANOVA, F(3,160) = 104, P = 1×10^−13^), with all light pulses resulting in at least 60% degradation of TIM. There is a weaker effect of increased intensity of the 10 min light pulses, 600 vs 7,000 nw/cm^2^, on TIM levels (two way ANOVA, F(1,80) = 4, P = 0.039). This could be due to a kinetic limitation from assaying TIM two hours subsequent to the pulses, or to a non-linearity between TIM degradation and phase shift amplitude.

Temporal integration in response to dim light phase shifts could act at the level of the CRY target TIM, or at a downstream step. Thus, we determined whether any of the neuronal classes showed a signature indicating that temporal integration is acting at the level of TIM degradation. For the equal photon light pulses, 600 nw/cm^2^ for 10 min vs 50 nw/cm^2^ for 120 min, there is a significant variation across all the neuronal classes (two way ANOVA, F(1,79) = 14, P = 3×10^−4^). Similar to the phase shift amplitudes shown in Figure 2, the longer yet dimmer light pulse shows a more severe effect on TIM degradation. This effect is enhanced in the core circadian neurons, the s-LNv's, 5th s-LNv, and the LNd's, as compared to the l-LNv's (two way ANOVA, F(1,84) = 11, P = 0.001). The l-LN_v_'s are not core circadian clock neurons, but instead mediate arousal and the direct effects of light on locomotor activity [27]–[29]. Within the core circadian neurons, the s-LNv class (including the 5^th^ s-LNv) shows significantly enhanced TIM degradation (two way ANOVA, F(1,62) = 12, P = 1×10^−3^), with a major effect occurring in the 5^th^ s-LNv(two way ANOVA, P = 6×10^−4^). This unexpected observation indicates that the 5^th^ s-LNv neuron may be particularly important for the process of photic temporal integration.

## Discussion

We find that the *Drosophila* CRY circadian photopigment is extraordinarily efficient at integrating photon information over long time periods extending to at least six hours. This finding aids understanding of the extraordinary light sensitivity of flies to 12∶12 hr LD entrainment [15], and provides a general means by which an inherently low-light sensitivity photopigment can achieve extraordinarily high effective light sensitivity.

Our results indicate that circadian photoreception via *Drosophila* CRY has unexpected similarities to the structurally distinct vertebrate circadian photoreceptor, melanopsin. Mouse melanopsin generates prolonged electrophysiological responses to single photons in the intrinsically photosensitive retinal ganglion cells (ipRGC's), yet the half-saturating response of the intrinsically photosensitive retinal ganglion cells requires 10^4^–10^6^ fold more photons than cones or rods, respectively [30]. This is largely due to the low pigment density of melanopsin in the ipRGC's relative to the opsin based photopigments in rods and cones, resulting in a low probability of photon capture. Thus, high effective light sensitivity must be generated via temporal integration over a time course of at least minutes [31], [32]. The same process holds for *Drosophila* CRY, though as we show above, efficient temporal integration can occur over time periods of hours. As with vertebrate melanopsin, dCRY is expressed in neurons lacking any specializations that would serve to concentrate the CRY photopigment. At ‘normal’ non-image forming protein concentrations, probability of photon capture per CRY molecule is low, potentially explaining the extremely high light levels required to visualize short term physiological responses [11], [20]. Thus, temporal integration appears to be a universal principle extending from arthropods to higher vertebrates as a mechanism to enhance physiological light sensitivity for non image forming light responses.

TIM degradation in the circadian pacemaker neurons shows a dose response relationship following limiting light intensity ZT20 light pulse induced phase shifts. This indicates that temporal integration is acting directly at the step of TIM degradation rather than at a downstream signaling step. Among the core circadian lateral neurons, the single 5^th^-s-LN_v_ neuron shows both the strongest effect of light, and the strongest effect of intensity duration reciprocity. The 5^th^-s-LN_v_, characterized by lack of PDF immunoreactivity, is distinguished from the other s-LN_v_ 's as a neuron that is part of a cluster of neurons more involved in evening rather than morning activity, and is thus more related to the ‘evening’ LN_d_ neurons [33]. The enhanced light sensitivity in the 5^th^-s-LN_v_ implies an important role in dim light detection for this neuron. Since CRY levels in the 5^th^-s-LN_v_ are not enhanced relative to other pacemaker neurons [34], some other component of the TIM degradation machinery must be more efficient in this neuron.

The described activities of *Drosophila* CRY have been observed at very high light intensities, ≥1 mw/cm^2^, ∼6 logs higher than the responses studied here, and ∼8 logs higher than required for dim light LD entrainment [15]. A particularly interesting activity of CRY is a light induced conformational change that persists in the dark with half-times of minutes to tens of minutes depending on oxygen concentration [19], [35]. Bright light exposure of CRY also leads to degradation of both TIM [36] and CRY itself [11], with CRY degradation occurring more slowly than TIM [11], [21] due to enhanced affinity of JET for TIM [14]. The CRY response to light is thus normally self-limiting, in that CRY itself can be degraded in a light dependent fashion. These pathways are at least partly distinct, as evidenced by dependence on distinct factors detected in a forward genetic screen [37].

The above studies in *Drosophila* could be misleading in several respects with regards to the dim light activity of CRY that we report. First, the high light intensities in which these studies were performed could hide differential light sensitivity of individual reactions. Second, many of the aforementioned studies were performed in cultured cells or in extracts from whole fly heads and thus could be misleading relative to CRY light dependent degradation reactions in the central circadian oscillator neurons of the fly. When examining whole brain extracts, the central oscillator neurons are so few in number that they are effectively hidden by the large number of peripheral CRY-containing neurons [17], and cultured cells that don't themselves have oscillatory capability may lack other factors found in central oscillatory neurons. Finally, the half-maximal light sensitivity of phase advancing light pulses that we measure is far higher than has been observed previously [16], [17], [38]. Part of the explanation is due to our use of monochromatic 466 nm blue light that is well matched to the action spectra of CRY [6], [21]. An additional explanation likely comes from our protocol for light/dark entrainment, in which we use relatively dim green light that is off the spectral maximum for CRY, yet is still able to efficiently entrain flies, presumably via contributions from visual photoreceptors. Bright light during entrainment can lead to destruction of CRY [34], with a half-time for recovery of 12–24 hrs. Thus, brain CRY levels during our light pulses are likely far higher than in previous studies. One could imagine that light-induced destruction of CRY could account for enhanced light sensitivity as durations increase and intensity decreases. However, we find no CRY degradation under the light pulse conditions used in this study (data not shown).

Light intensity is an under-appreciated variable in behavioral studies, with many studies neglecting to measure or mention these values. Our studies, performed at limiting light levels, are showing the importance of being aware of intensity as a critical variable. That *Drosophila* CRY and vertebrate melanopsin use a similar dim light integration mechanism indicates a striking example of an evolutionarily convergent solution to the problem of dim light detection in non image forming neurons.

## Materials and Methods

The dim light source is 5050 RGB Waterproof SMD Flexible LED Strips, 30 LEDs/meter, currently available from several suppliers on eBay. Each LED consists of separately addressable RGB LEDs, with sets of three LED connected in series with internal resistors, & groups of three then connected in parallel. Maximal output of the blue LED is at 466 nm, and the green LED is at 521 nm (Figure S1) as measured with a spectrophotometer (Ocean Optics, USB4000). LED strips were cut to ∼18″ lengths and mounted behind a light diffusing opaque plexiglass plate. Intensity was controlled by varying the voltage supplied to the LEDs, and light intensities measured as detailed in [15].

Light pulses were given to flies entrained for several days to 2 µw/cm^2^ green light (5.2×10^6^ photons/cm^2^/sec) from the LED strips, using green light to limit possible photopigment degradation [34]. Pulses were given on the evening of the last day of light.

### Fly strains

The null alleles *cry^02^* [39] and *norpA^P41^* [23] were obtained from the laboratory of Herman Wijnen. Since both strains are in a *w* mutant background, the *w^1118^* strain was used as a wild type control. Sequence analyses (data not shown) show that each of these strains contains the s-tim allele [40].

Flies were housed in Trikinetics (Waltham, MA) 5 mm activity monitor tubes in light tight coffins in a room controlled to ∼21C and 55–60% relative humidity. Phase changes following light pulses were assayed by detecting computer called activity off times using Clocklab software (Actimetrics Corp, Wilmette, IL). Phase shift data was accumulated on individual flies. Statistics were determined by ANOVA with post-hoc adjustment for multiple comparisons.

### Immunofluorescence

Whole flies were fixed for 2.5 hrs at room temperature in 4% PFA with 0.1% Triton X-100, followed by four 10 min washes in PBS (130 mM NaCl, 7 mM Na2HPO4, 3 mM KH2PO4), then stored in fresh PBS+0.01% NaN_3_.

Brains were dissected and washed in PBT (PBS with 0.1% BSA and 0.3% Triton X-100) 2×5 min, then incubated overnight with primary antibodies diluted in PBT. This was followed by three 5 min and two 30 min washes in PBT with 1% goat serum, 6 hr incubation in secondary antibodies diluted in the same solution, and then brains are washed in PBT four times over a 24 hr period, followed by a final wash in PBS prior to mounting in Vectashield mounting medium (Vector laboratories).

### Antibodies

Here we used rat anti-Tim (1∶1000; kindly provided by Jadwiga Giebultowicz) & mouse anti-PDF antibody (1∶1000; Developmental Studies Hybridoma Bank, University of Iowa), secondaries are goat anti-mouse (PDF) IgG conjugated to AlexaFluor 488 (Molecular Probes) and goat anti-rat (Tim) IgG conjugated to Cy3 (Jackson ImmunoResearch).

Confocal microscopy was performed with a Fluoview300 (Olympus).

### Quantitation

The quantification of the TIM staining intensity was performed on single confocal images with ImageJ (http://rsbweb.nih.gov/ij/) as previously described [41]. PDF immunostaining was used to identify LNv's. First we chose the appropriate slice within stack for each neuron, then selected ROI based on PDF staining (except for LNd's & the 5th s-LNv), and measured mean pixel intensity of TIM staining. To compensate for background, the mean pixel intensity surrounding the neuron was subtracted. The compensated values were averaged for all neurons of a given class. We used one hemisphere per brain for the quantification and calculated mean ± SEM from 11 hemispheres of 11 brains. We could not measure any bleed through from intense PDF positive neuronal projections into the TIM channel.

## Supporting Information

Figure S1 Illuminance spectra of the blue and green light sources used in this manuscript. Spectra were traced from output of an Ocean Optics USB4000 spectrophotometer.
(TIFF)

Figure S2 A repeat of Figure 3C using *cry^02^* except each light pulse is at 10× (A) or 100× (B) the light intensities used in Figure 3C. N.D.: Not done. Neither figure shows significant changes as a function of light pulse intensity relative to the no pulse control: (A) P>0.19; (B) P>0.24. For each experiment, n = 16–20 light pulse condition. Error bars = SEM.
(TIFF)

Table S1 P Values for Two way ANOVAs in Figure 6; Comparisons of 600 nw/cm^2^ 10 min & 50 nw/cm^2^ 120 min TIM level datapoints within & between cell types. Two way ANOVA calculations as per Materials & Methods.
(TIFF)
