# Supplementary figures and images for: Exquisite Light Sensitivity of Drosophila melanogaster Cryptochrome
Source: PLoS Genet. 2013 Jul 18;9(7):e1003615. doi: 10.1371/journal.pgen.1003615 (PMC3715431; doi:10.1371/journal.pgen.1003615)

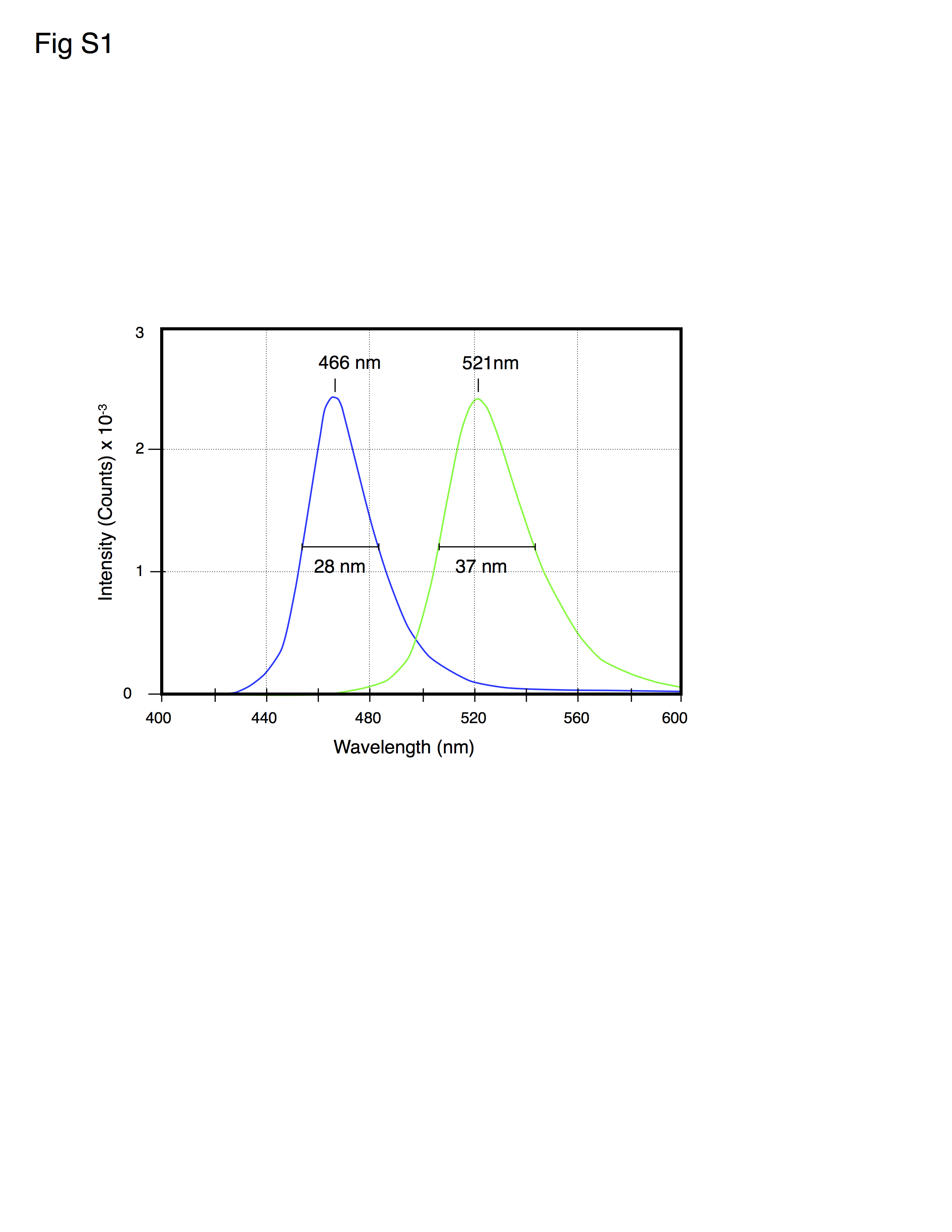

Supplement: Figure S1 — Illuminance spectra of the blue and green light sources used in this manuscript. Spectra were traced from output of an Ocean Optics USB4000 spectrophotometer. (TIFF) [file pgen.1003615.s001.tiff]

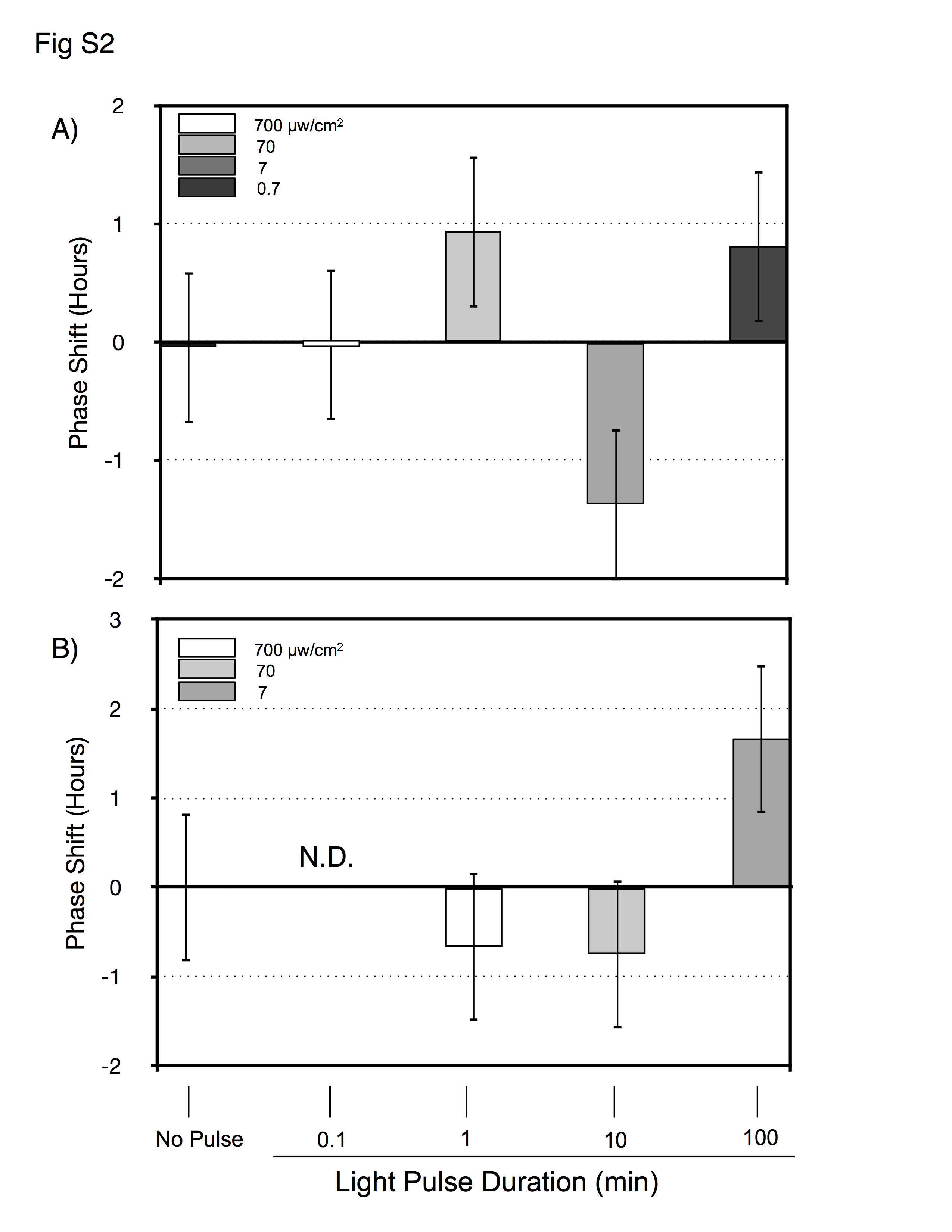

Supplement: Figure S2 — A repeat of Figure 3C using cry02 except each light pulse is at 10× (A) or 100× (B) the light intensities used in Figure 3C. N.D.: Not done. Neither figure shows significant changes as a function of light pulse intensity relative to the no pulse control: (A) P>0.19; (B) P>0.24. For each experiment, n = 16–20 light pulse condition. Error bars = SEM. (TIFF) [file pgen.1003615.s002.tiff]

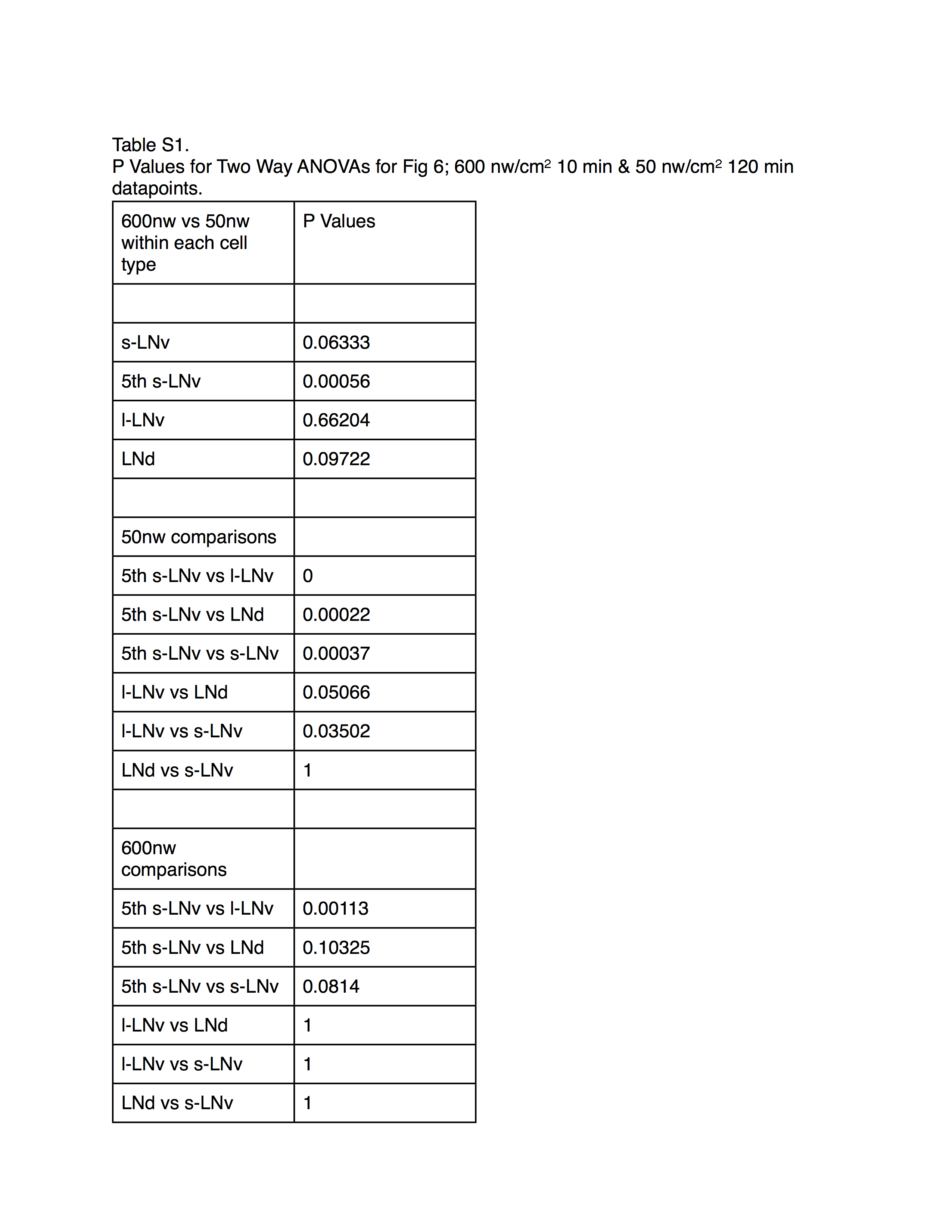

Supplement: Table S1 — P Values for Two way ANOVAs in Figure 6; Comparisons of 600 nw/cm2 10 min & 50 nw/cm2 120 min TIM level datapoints within & between cell types. Two way ANOVA calculations as per Materials & Methods. (TIFF) [file pgen.1003615.s003.tiff]
